# Evaluating the national multisite implementation of dialectical behaviour therapy in a community setting: a mixed methods approach

**DOI:** 10.1186/s12888-020-02610-3

**Published:** 2020-05-14

**Authors:** Daniel Flynn, Mary Joyce, Conall Gillespie, Mary Kells, Michaela Swales, Ailbhe Spillane, Justina Hurley, Aoife Hayes, Edel Gallagher, Ella Arensman, Mareike Weihrauch

**Affiliations:** 1 Cork Mental Health Services, Cork Kerry Community Healthcare, Health Service Executive, St Finbarr’s Hospital, Cork, Ireland; 2 National Suicide Research Foundation, University College Cork, Western Gateway Building, Cork, Ireland; 3 Cork Mental Health Services, Cork Kerry Community Healthcare, Health Service Executive, Inniscarraig House, Western Road, Cork, Ireland; 4 Betsi Cadwaladr University Health Board & North Wales Clinical Psychology Programme, School of Psychology, Bangor University, Bangor, Wales; 5 National Suicide Research Foundation and School of Public Health, University College Cork, Western Gateway Building, Cork, Ireland

**Keywords:** Dialectical behaviour therapy, Implementation, Evaluation, Community settings, Public health service, Borderline personality disorder, Team leaders, DBT therapists

## Abstract

**Background:**

The implementation of evidence-based interventions for borderline personality disorder in community settings is important given that individuals with this diagnosis are often extensive users of both inpatient and outpatient mental health services. Although work in this area is limited, previous studies have identified facilitators and barriers to successful DBT implementation. This study seeks to expand on previous work by evaluating a coordinated implementation of DBT in community settings at a national level. The Consolidated Framework for Implementation Research (CFIR) (Damschroder et al., Implementation Sci. 4:50, 2009) provided structural guidance for this national level coordinated implementation.

**Methods:**

A mixed methods approach was utilised to explore the national multisite implementation of DBT from the perspective of team leaders and therapists who participated in the coordinated training and subsequent implementation of DBT. Qualitative interviews with DBT team leaders (*n* = 8) explored their experiences of implementing DBT in their local service and was analysed using content analysis. Quantitative surveys from DBT therapists (*n* = 74) examined their experience of multiple aspects of the implementation process including orienting the system, and preparations and support for implementation. Frequencies of responses were calculated. Written qualitative feedback was analysed using content analysis.

**Results:**

Five themes were identified from the interview data: team formation, implementation preparation, client selection, service level challenges and team leader role. Participants identified team size and support for the team leader as key points for consideration in DBT implementation. Key challenges encountered were the lack of system support to facilitate phone coaching and a lack of allocated time to focus on DBT. Implementation facilitators included having dedicated team members and support from management.

**Conclusions:**

The barriers and facilitators identified in this study are broadly similar to those reported in previous research. Barriers and facilitators were identified across several domains of the CFIR and are consistent with a recently published DBT implementation Framework (Toms et al., Borderline Personal Disord Emot Dysregul. 6: 2, 2019). Future research should pay particular attention to the domain of characteristics of individuals involved in DBT implementation. The results highlight the importance of a mandated service plan for the coordinated implementation of an evidence-based treatment in a public health service.

**Trial registration:**

ClinicalTrials.gov ID: NCT03180541; Registered June 7th 2017 ‘retrospectively registered’.

## Background

Advancement in the treatment of, and recovery from, mental health disorders requires integration of evidence-based practice (EBP) into community mental health services [1]. The implementation of EBP for borderline personality disorder (BPD) in community settings is especially important as individuals with this diagnosis are amongst the most extensive users of inpatient and outpatient mental health services [2–4]. Additionally, the treatment of individuals with BPD is often described as complex [5, 6] and costly [7].

Treatments such as dialectical behaviour therapy (DBT [8–10]), schema therapy [11], mentalisation-based therapy [12], and transference-focused psychotherapy [13] have been developed for treating BPD. DBT is the most researched treatment option with more than a dozen randomised controlled trials (e.g. [14–16]) which have investigated its efficacy at multiple independent sites within different healthcare infrastructures and different countries [17, 18]. Participation in DBT has shown improved outcomes for individuals; specifically reductions in suicidal behaviour, suicidal ideation, BPD symptoms, depression, and health service utilisation [15, 19, 20].

In addition to efficacy, DBT has demonstrated effectiveness in routine clinical settings. Comtois and colleagues [21] were the first to evaluate the effectiveness of DBT in a community mental health setting. Their study and subsequent effectiveness evaluations (e.g. [22–25]) have reported positive outcomes for individuals with BPD who participate in community based DBT programmes. In line with this, best practice guidelines for the delivery of evidence-based interventions in mental health services also recommend DBT for the treatment of BPD in community settings (e.g. [26, 27]).

The transfer of EBP from research into an existing healthcare service can be challenging and time-consuming, and requires persistence [28, 29]. Studies which have explored implementation of EBP in general healthcare settings [30, 31] report similar implementation facilitators and barriers to those which focus on DBT. Interview data from DBT clinicians has identified several barriers to successful implementation of DBT programmes in community settings such as lack of organisational support for the intervention, inadequate planning for programme implementation, competing therapeutic priorities within the service, attrition of trained staff and insufficient protected time to deliver full DBT [17, 32, 33]. Facilitators of successful DBT implementation include organisational support (provision of funding for training and supervision; ensuring therapists have protected DBT time), team cohesion, skill and leadership, and observation of positive clinical outcomes [32–36]. Investigations into the sustainability of DBT programmes in the UK National Health Service (NHS) demonstrate that programmes are vulnerable to closure in the first 5 years after formation [32, 33] with data from Ireland indicating a similar pattern [37]. Successful and sustainable DBT programmes require implementation planning, especially to assess whether DBT aligns with organisational goals and whether the available resources are sufficient to provide DBT alongside existing interventions [17, 32, 38, 39]. Improving organisational-level support through education [17, 40], carefully selecting staff for training, and providing training on an ongoing basis to counter staff attrition is also crucial [32, 35]. Finally, monitoring intervention effectiveness, communicating results back to stakeholders, and providing an environment to foster team communication, cohesion and supervision are important considerations in DBT implementation [32, 36]. A recent review of DBT implementation literature endorsed the importance of assessing for ‘goodness of fit’ between DBT and the organisation; effective leadership or ‘championing’ of the DBT team; and recruitment of therapists with sufficient cognitive flexibility and non-judgemental attitudes who are supported with ongoing supervision and training [34].

In Ireland, policy guidelines for the provision of mental health services in the national public health system recommend DBT as an evidence-based treatment for individuals with BPD [27]. In the absence of a national mandate for the implementation of DBT in community settings, teams interested in DBT training have primarily sought funding from the National Office for Suicide Prevention (NOSP) which was established within the Irish public health service in 2005 to tackle high rates of suicide and self-harm in Ireland [41]. In 2013, as requests for funding for DBT training from individual teams had begun to increase, a proposal for a coordinated approach to implementation of DBT at a national level was put forward to the NOSP. The proposal was initiated by a Clinical Psychologist who had experience as a DBT therapist and team leader, had implemented DBT in their local service, and subsequently expanded the DBT service to a wider geographical region following initial implementation success [42]. A coordinated approach to implementation allowed for consideration to known facilitators and barriers in an attempt to maximise successful DBT implementation in community mental health settings in Ireland. The National DBT Project Ireland was subsequently established with an accepted funding proposal to initially train 16 teams (two cohorts of eight teams) in both adult and child and adolescent mental health services over a 2 year period. Funding was also allocated for a team to coordinate and support the multisite implementation and evaluation across multiple domains (effectiveness, economic and implementation evaluation) [42].

There are a number of implementation models and frameworks which can be utilised when considering implementation of an evidence-based intervention such as DBT. As this project involved implementation at a national level, the framework that was deemed most relevant at the time and provided a structural guidance for implementation within the Irish public health system was the Consolidated Framework for Implementation Research (CFIR) [43]. The CFIR offers an overarching typology to assist with identifying what works where and why across multiple contexts; thus, it has the capacity to structure implementation at a macro level. The CFIR comprises five major domains: the intervention, outer setting, inner setting, the individuals involved and the process of implementation. By considering the various domains of the CFIR, previously identified barriers and facilitators to DBT implementation could be given due attention for this national multisite implementation of DBT in a community setting (a detailed outline of how this was done can be found in [42, 44]). In particular, the CFIR ‘Process’ domain which involves planning, engaging, executing and reflecting/evaluating was followed in an iterative manner throughout the project.

The National DBT Project Ireland was the first to coordinate and evaluate DBT training at a national level and the first to develop a protocol to consider known facilitators and barriers for a coordinated implementation of DBT using the CFIR.

## Methods

### Aim, design and study setting

The aim of this study was to evaluate a coordinated implementation of DBT in community settings at a national level. This mixed methods study reports on the experiences of DBT team leaders and therapists who participated in the coordinated training and subsequent implementation of DBT in Ireland. A mixed methods approach was chosen to explore and examine the national multisite implementation of DBT from the perspective of participating clinicians. The current study employed a sequential mixed methods design which comprised of two phases of evaluation. Phase one involved qualitative interviews with the first cohort of DBT team leaders (*n* = 8). The interviews aimed to explore participants’ experience of DBT implementation in their service. The second phase of the study involved the distribution of a survey to all DBT therapists who completed training via the National DBT Project Ireland (NDBTPI). The survey measured therapists’ experiences of various aspects of the implementation process.

The setting for this study was community based mental health services within the national public health system in Ireland (a detailed overview of this health system structure can be found in [42]). While there is national oversight of mental health service provision in this health system, there is local variation in form and function of the numerous multi-disciplinary mental health teams which operate within this broader network. Each team reports to their own local governance structure where there is variation in service provision dependent on populations, staffing resources and local priorities, but where teams are guided by a national policy document [27]. DBT teams in community settings in Ireland typically consist of core multi-disciplinary staff from multiple community mental health teams (CMHT) who are seconded from their multi-disciplinary CMHT to train in DBT.

Optimised DBT programmes require implementation planning where the intervention is aligned with organisational goals and staff are carefully selected and supported to train in and provide the intervention on a sustained basis. Supervision, ongoing training, monitoring of outcomes, and clear communication to stakeholders across all levels of an organisation are required to maximise the likelihood of successful implementation in a public mental health system [2, 34, 36–37). The current study considered all of these factors under the CFIR framework for the coordinated implementation with a particular emphasis on the ‘Process’ domain (see Additional file 1).

### Participants

Participants in this study were clinicians who completed DBT training via the NDBTPI. DBT training was provided by a licensed training provider to ensure both high quality and consistency of training across teams. In phase one of the study, all eight team leaders from the first cohort of teams that completed training were recruited. Participants were four team leaders from DBT teams in adult mental health services (AMHS) and four team leaders from DBT-A teams in child and adolescent mental health services (CAMHS) (*n* = 8). In phase two of the study, all therapists (*n* = 123) from the sixteen teams that trained in cohorts one and two of the project were invited to participate in the study. Survey data was available for 74 participants (60%). Reasons for missing data included participants having left the DBT team or their local service, or participants not returning the survey. As the data provided by participants in phase two of the study was anonymous in an attempt to reduce response bias, demographic information was not collected from DBT therapists.

### Measures

#### Phase 1 - qualitative interview

A structured interview schedule was developed for the study which covered four main topics related to participants’ experiences of DBT implementation: 1. the process of introducing the DBT model to local services and orientating the service/local management; 2. preparations for the implementation of local DBT programmes; 3. team leader’s experiences of delivery of the DBT programme and their experience as DBT team leader; and 4. support for the implementation of the DBT programmes from local management. The four topics and corresponding interview questions are presented in Table 1. The interview schedule was developed by members of the NDBTPI research team and was drawn up by reviewing previous literature on the topic. The content of the interview schedule was refined through input from members of the NDBTPI Research Advisory Group including a DBT expert, two experienced DBT clinicians and an expert in self-harm and suicide research.

**Table 1:** Structured interview schedule

**Section 1: Introduction of DBT and orientating service to DBT model**	
1. How well did DBT fit with your existing service structures and policies? What facilitated the process?	
2. How were the DBT team leaders and team members identified? Any considerations for team size?	
3. Did you have to take specific steps to guide the service in preparing to train for and implement DBT?	
4. What would you have done differently/advise another team going forward?	
5. What other challenges (if any) did your team face when introducing the DBT model to your service?	
**Section 2: Preparations for implementing DBT in your local service**	
6. What steps were taken to orient the service for the implementation? What worked best?	
7. What would you have done differently/advise another team going forward?	
8. How did the team prepare for part one of training? Any difficulties encountered before/after?	
9. Were there any specific learnings in implementation that would be helpful for future teams?	
10. Did you encounter any difficulties in the identification of suitable clients for the programme?	
**Section 3: Experiences of delivering the DBT programme and as DBT team leader**	
11. Did you encounter any difficulties in the delivery of the programme? Any learnings from these?	
12. What challenges (if any) have you faced in your role as team leader? Any supports required?	
13. What are the key qualities that a DBT leader should possess for implementing DBT?	
**Section 4: Support for the implementation of the DBT programme**	
14. Was administrative support available for non-clinical elements of the DBT programme? Did the team feel supported in this?	
15. Was organisational support from local management available? Did the team feel supported in this?	
16. Any progress reports to local management regarding the implementation of the programme?	
17. Any difficulties in obtaining necessary time to prepare for and deliver your DBT programme?	

#### Phase 2 - therapist survey

As there was no previously developed valid measure to assess clinicians’ experience of DBT implementation, a quantitative survey (see Additional file 2) was developed by the research team to explore various aspects of the implementation process. Analysis of the qualitative interview data from phase one of this study, in addition to a review of relevant literature, informed the identification of topics to be included in the survey. Survey topics included participants’ experience of being part of a coordinated implementation of DBT, perceived barriers and facilitators to DBT implementation, and participants’ experiences of DBT training and supervision. Participants rated their experiences on each of these aspects of implementation on a Likert type scale. Participants were also provided with an opportunity to include written qualitative feedback on various aspects of the implementation such as identifying potential supports to aid long-term sustainability of DBT in their service and other supports they felt might be beneficial for therapists and teams.

### Procedure

Given that there were multiple independent sites for this study, each with their own ethics committee, ethical approval to conduct this study was obtained at each individual site. All therapists who completed training via the NDBTPI were invited to participate in the research study for a two-year period to contribute to the research evaluation at various time-points. Participants were informed that study participation would require provision of feedback on aspects of DBT implementation within their service.

In phase one, DBT team leaders from the first cohort of teams who completed DBT training were invited to participate in a telephone interview with a member of the research team. Telephone interviews took place in October 2014, 10 months after attending DBT Intensive Training Part 1.^1^ By then, all teams had completed Intensive Training Part 2 and participants from DBT teams in AMHS had delivered each of the skills modules at least once (i.e. had at least 6 months of a standard DBT programme delivered) while participants in CAMHS had implemented at least one full 16-week DBT-A programme. Interviews were conducted with a member of the research team^2^ and lasted approximately 1 h. All interviews were audio-recorded and transcribed in preparation for analysis. Pseudonyms were assigned to all interview data and no identifying information was included in the transcribed interviews.

In phase two, surveys were distributed to all DBT therapists who had attended DBT training via the NDBTPI. The surveys were posted to the team leaders of all 16 teams who trained via the project. Team leaders then distributed the surveys at the DBT team consultation meeting. Participants completed the survey anonymously and returned their completed survey in a sealed envelope to their team leader. The team leader subsequently returned completed surveys to the research team via post. Surveys were returned from all 16 teams. Data collection occurred at three time-points: prior to attending Intensive Training Part 1^1^; 6 months after Part 1; and 2 years after Part 1. Data from the final data collection time-point (December 2015 for Cohort 1 and September 2016 for Cohort 2) will be focused on for the purpose of this study.

### Data analysis

A sequential analytic strategy was employed which involved qualitative analysis followed by quantitative analysis (qual → QUAN). The qualitative interviews were transcribed verbatim and analysed using a content analysis approach which comprises identifying codes and transferring them into significant themes. A conventional approach to content analysis was utilised, the aim of which is to describe a phenomenon [45]. The first step of the analysis process was to become fully immersed in the data through reading and re-reading of the transcribed interviews. This was followed by the generation of codes using exact words and quotes from the data to capture key concepts. The third step of the analysis involved a review of the researcher’s first impressions of the data, thoughts and initial analysis. Labels were next identified for codes that were reflective of more than one key thought. The final step involved sorting the codes into categories based on how they were related and linked. Two authors (MJ, CG) independently coded and analysed the eight interviews. The findings were then reviewed whereby the authors came together to discuss the results of the independent analyses and ensure agreement on the themes identified.

For the quantitative element of the therapist survey (*n* = 74), frequencies of responses were recorded based on the participants’ ratings on the different survey items. Analysis of written qualitative feedback from the therapist survey was conducted independently by two of the authors (CG, EG) using summative content analysis [45]. This analysis first involved identifying and quantifying specific words and content in the text to gain an understanding of the contextual use of the words. Frequencies of responses within the qualitative data were recorded with this approach. An interpretation of the content was then carried out to identify underlying meaning of the responses.

## Results

### Phase 1 – qualitative interviews

Five main themes were identified from the analysis process which illustrated challenges at different points of the implementation process and included advice for future DBT teams: ‘team formation’, ‘implementation preparation’, ‘client recruitment’, ‘service challenges’, and ‘team leader role’.

#### Theme 1 – team formation

The first theme relates to team member selection and team size. Of the eight teams who trained with the project, seven teams self-selected to attend Intensive Training. Team member selection for the remaining team was primarily decided upon by management. Participants discussed the importance of considering team size and associated advantages/disadvantages. At the time of DBT training for this cohort of teams (2013), a minimum of four therapists were required to establish a new DBT team while the maximum number was ten. Challenges identified with having a larger team included time pressure in the weekly team consultation and an uneven distribution of work within the team:

*“There is this sense that if you are a part of a big team, you know, the bystander effect – it’s somebody else’s responsibility to do that”.* (Participant 5)

Team dynamics was sometimes a challenge in larger teams. While participants from smaller teams reported strong team cohesion, there was also the challenge of having the required resources available to offer all modes of treatment with a smaller number of therapists.

#### Theme 2 – implementation preparation

The second theme highlighted the importance of various tasks completed by teams in preparation for the implementation of DBT in their service. A significant amount of groundwork was completed prior to implementation with six participants reporting that they delivered a presentation on DBT to management, Consultant Psychiatrists and their local multi-disciplinary teams. The presentations typically provided general information on the DBT programme as well as specific information about the referral process and eligibility criteria. Participants felt that the presentation provided the DBT team with the opportunity to *“[sell] the programme both at management and at grass roots level”* (Participant 3). The presentation also facilitated open discussions with other health professionals, in particular with Psychiatrists, so they *“knew who we were, what we did, and who was appropriate and who wasn’t appropriate, that was critical”* (Participant 3).

The opportunity to link with and get advice from an existing team was also identified as useful when preparing for DBT implementation. Of the eight participants, three reported having an opportunity to do this, while another two participants recommended this to future teams in their preparation for implementation. The teams that had an opportunity to meet with a pre-existing team found it useful in terms of identifying potential barriers to implementation and access to resources:

*“I found that really helpful...they were able to kind of go through any teething problems they had, any difficulties, and then by looking at the difficulties that they had encountered I suppose we were able to prevent ourselves having similar difficulties and we were able to see what worked for them that could maybe work for us”* (Participant 1)*.*

#### Theme 3 – client selection

The third theme related to the selection of suitable clients for the DBT programme. Seven participants identified challenges related to client selection including client commitment issues, the duration of the pre-treatment phase and rushed recruitment. The latter was a particular issue for some participants given the requirement that their DBT programme have commenced before Intensive Training Part 2 took place:

*“we did our part one training and then it was kind of said to us you know you need to have this programme up and running…..by the time you do part two”.* (Participant 5)

Participants recommended giving more time for screening and pre-treatment (more than 4 weeks) in order to determine suitability of clients and ensure commitment:

*“I suppose it’s really starting the referral process well in advance of the programme, em I think realistically it may take like 2 or 2 and half months to identify someone...I think… be willing to let the client go during the pre-treatment stage. If you’re too invested in actually getting the client on for the wrong reasons, because nobody else is coming on, that really throws up a lot of problems later on in the programme.”* (Participant 2)

#### Theme 4 – service level challenges

All eight participants identified various challenges to DBT implementation in their service including issues around referral eligibility criteria, phone coaching and lack of back-fill for existing workload. With regard to referral criteria, most of the adult DBT teams encountered issues with some initial multi-disciplinary team reticence in the use of borderline personality disorder or emotionally unstable personality disorder as a diagnostic label:

*“I think the consultants were quite reluctant to put, give the diagnosis of personality disorder to clients and I think that kind of came up initially at the start and the referrals were quite slow...”* (Participant 1)

While the issue of diagnostic labels emerged as a challenge for participants working in AMHS, this was not the case for participants working in child and adolescent services as diagnostic labels for BPD are not typically utilised in CAMHS in Ireland.

Issues with phone coaching were identified in six teams with participants reporting therapist reluctance to provide out of hours phone coaching, no time-off-in-lieu given for out of hours phone cover, lack of management support for phone coaching, and an issue regarding clinical responsibility for DBT clients outside of core work hours. In considering these challenges, participants noted that individual therapists on their teams set personal limits with regard to the provision of phone coaching:

*“we couldn’t offer it uniformly as a service, so some people did, as an individual negotiation between them and their client, but as a service, we offer nine to five during working days, typically Monday to Friday.”* (Participant 3)

Five participants felt that balancing the demands of DBT with the other aspects of their clinical work was difficult. Although teams had received support to commit 1.5 days per week for the provision of DBT at the training application stage, the reality was that many therapists still had to manage their pre-existing workload in addition to the allocated time for DBT.

*“Well you see that’s the thing it’s not really a day and a half if you’re actually still doing everything else with it…you know we were sticking it in our diaries but then you’re squashing everything into the rest of the time that you usually would have a whole week to do.”* (Participant 4)

Other challenges to implementation under this theme included: lack of administrative support such as preparing and developing materials (*n* = 5); staff being pulled from weekly consultation (*n* = 4); logistical or practical challenges (e.g. obtaining room space for treatment delivery; *n* = 4); and resistance from staff outside of the DBT team given the time commitment required by DBT trained staff to develop and implement the programme (*n* = 3).

#### Theme 5 – team leader role

The fifth theme referred to the role of the team leader and the accompanying responsibility and commitment. Participants noted that there was additional pressure and stress accompanying the role of team leader; five participants reported that their DBT work required more than the allocated 1.5 days. Half of the participants (*n* = 4) also highlighted that the DBT team leader needs to have the ability to manage team dynamics and conflict within the team:

*“...it was about trying to manage those different difficult dynamics coming up, and I think being aware of different, kind of, interpersonal kind of conflicts and how to manage them is something you need to be able to do.”* (Participant 1)

Strong interpersonal and communication skills, organisation and delegation skills, and the ability to keep the team motivated were other important qualities identified for success in the team leader role. In addition, four participants stated that a team leader needs to look beyond the everyday work of DBT and look to the future in terms of planning logistics and governance:

*“I also think keeping an eye on the bigger picture all the time, like I feel I’m constantly aware of a step ahead of what’s happening.”* (Participant 7)

Two participants also recommended having additional support for team leaders such as individual supervision or a team leader forum to help guide them on challenges associated with their role.

### Phase 2 - therapist surveys

The findings from phase one informed the content of the surveys distributed to the therapists in phase two of the study. Participants (*n* = 74) completed a survey which comprised several aspects of implementation including participants’ experience of the coordinated implementation approach, impact of expert supervision, facilitators and barriers encountered, and the impact of DBT Foundational Training on their service.

#### Experience of the coordinated implementation

Participants were first asked about their experience of participating in a coordinated implementation of DBT. Almost half of the sample (46%; *n* = 34) felt additional information/assistance could have been provided by the NDBTPI coordinating team to help with the implementation of DBT in their area. The most common suggestions put forward by participants include increased liaison from the DBT coordinating team with health service senior management (*n* = 6), the organisation of days where DBT teams could meet and share ideas (*n* = 6), and the provision of more I.T. resources and materials (*n* = 5).

Seventy-six percent of participants (*n* = 56) indicated that additional training would be helpful for therapists/teams to assist with long-term sustainability of DBT in their service. Suggestions for types of training were put forward by 37 participants with the most common occurring proposals including booster and refresher training, group skills workshops or training in allied DBT informed programmes such as Family Connections. Participants also identified that liaising and meeting with other DBT teams to share/discuss difficulties and experiences (*n* = 18), ongoing supervision (*n* = 17) and the need to train new staff (*n* = 15) to combat staff attrition and turnover would be helpful for long-term sustainability.

#### Supervision

All participants rated supervision to be either ‘very helpful’ or ‘somewhat helpful’ in their practice regarding the programme elements of DBT (skills group, individual therapy, phone coaching, team consultation). In particular, participants found supervision helpful with regard to advice and adherence to the programme. In terms of structuring the environment or service related issues, 78% of participants found supervision either ‘very helpful’ or ‘somewhat helpful’ for their DBT practice. Given that supervisors were based in jurisdictions outside of the Irish public health system, some participants found advice relating to local service issues less helpful when compared to programme elements advice. Participants highlighted issues with I.T., noting that many local services did not support video calls. Therefore, teams were limited to using telephone conference calls for communicating with supervisors. In addition, systemic I.T. difficulties meant there were issues when trying to send and share therapy recordings with supervisors.

In terms of sufficiency of supervision resources made available, 68% of participants found the allocation by the NDBTPI to each team to be sufficient, 23% of participants did not, and 9% of participants did not specify. When asked to provide further detail about the supervision resource, three main themes were identified with participants preferring a more face-to-face supervision interaction, a requirement for more regular supervision, and the importance of sustaining supervision in the future.

A total of 78% of participants reported that additional support could be provided regarding supervision opportunities to help with long-term sustainability of DBT in their service. When asked to elaborate, participants identified ongoing supervision and training of local supervisors as two key items that would be helpful.

#### Facilitators and barriers in implementing DBT

Having personally invested, highly dedicated team members was identified as the main facilitating factor for the implementation of DBT programmes (*n* = 26). Over a quarter of participants (*n* = 18) noted that support from local management and the wider community team was the most facilitating factor for DBT implementation. Other identified facilitators included having an effective team leader, supervision and training (see Table 2).

**Table 2:** Identified facilitators and barriers to DBT Implementation and examples of participants’ responses

Themes	Participants response examples	Number	Percent
*Facilitators*			
Dedication of team members	“Motivated clinicians willing to go above and beyond the call of duty”	26	38.3
Support from management and wider team	“Support from management, understanding of service need for this therapy”	18	26.5
Having an effective Team Leader	“A strong dedicated team-leader, who is a definite believer and advocate of DBT and generous with her time in supporting the team and project”	9	13.2
Supervision	“...external supervision has increased motivation”	7	10.3
Training	“The two week training provided a strong immersion in DBT”	7	10.3
*Barriers*			
Lack of support from management	“Management not providing infrastructure, fighting for room space to run programme...”	28	41.2
Logistical challenges	“Difficulty in finding appropriate space to accommodate numbers of DBT clients attending” “Lack of monetary support for equipment and refreshments etc.”	15	22.1
Time commitment and balancing of other roles	“Lack of cover for caseload while implementing DBT meaning need to carry previous load as well as DBT increasing possibility of burn-out”	12	17.7
Staffing resources including attrition	“Clinicians who are DBT trained have left the service which has made it more difficult to successfully implement DBT”	9	13.2

Perceived barriers to the implementation of DBT programmes included lack of management support (*n* = 28) and logistical challenges including lack of space, geographical factors impacting client access to DBT and a lack of resources for programme delivery (*n* = 15). Time commitment and staffing resources were further implementation barriers identified (see Table 2).

#### DBT Foundational Training

DBT Foundational Training is a five-day training for clinicians who wish to join an existing Intensively Trained DBT Team. This training was offered to DBT teams who had trained via the NDBTPI on an annual basis. When participants were asked to qualitatively describe the impact of additional therapists joining their team, four main themes were identified: 1. increased capacity to deliver DBT services, 2. new perspectives, 3. maintaining the team, and 4. the challenge of new team dynamics. Participants were then asked what additional supports, if any, would help to facilitate the integration of new team members in their DBT team. Participants highlighted the importance of supervision and additional support in the initial stages, in particular guidance and direction on how to support and train up new team members, and support with navigating team dynamics in the context of changing membership.

## Discussion

This mixed methods study is the first of its kind to evaluate and report on a coordinated national implementation of DBT. While previously identified facilitators and barriers were considered in the design phase of this national implementation approach, some key challenges prevailed. In addition, although results from this study are broadly in line with previous research on DBT implementation, findings from the qualitative component of this study highlight additional factors that warrant consideration. These include team formation, client selection and the role of the team leader. We believe that these additional factors are relevant for individual teams who form and train independently, as well as those who train as part of a coordinated implementation effort.

The quantitative component of the study identified that some therapists, having trained as part of a coordinated implementation project, felt that further information and assistance could be provided by the coordinating team to aid implementation. Having personally invested, dedicated team members was reported as the primary facilitator to implementation while lack of support from management was identified as the chief barrier.

The constructs outlined in the CFIR provide a foundation for understanding implementation and what aspects help or hinder the process across different contexts [43]. The qualitative and quantitative results have been integrated in this discussion section where the key findings are presented in the context of the relevant CFIR domains.

### The intervention

The first construct of the CFIR focuses on the characteristics of the intervention itself. Relevant to the intervention delivered in this study, phone coaching is a conceptually unique component of DBT which posed a distinct intervention-related challenge for therapists. Phone coaching is a vehicle to support clients in generalising skills learned in the clinical setting and apply them effectively in their daily life [46]. In principle, it is available as needed by clients. A challenge for a publicly funded health system, which routinely operates between 09.00 and 17.00, is how to support therapists and systems in working outside standard clinical hours to offer this modality of treatment. No formal mandate for the provision of phone coaching was in place prior to this coordinated implementation effort. This may have, in part, contributed to participants reporting apprehension about phone coaching. Participants expressed concern about holding clinical responsibility for a DBT client in the context of phone coaching when heretofore; clients would have been encouraged to attend out-of-hours emergency services if in emotional distress. Phone coaching is an essential element of DBT with evidence to suggest that more frequent phone contact is associated with a decrease in dropout and psychological symptoms, and an increase in client and therapist satisfaction [47]. While the NDBTPI coordinating team encouraged therapists to work with local management and use supervision to guide practice, the coordinating team did not have the authority to mandate change for individuals or systems in their practice.

Related to the intervention itself was the provision of expert supervision which was noted by many participants as an important facilitator to successful DBT implementation. Previous research has also identified that this ongoing consultation is important for DBT implementation success [17, 36, 48, 49]. It is hoped that the external support received during supervision would enhance therapist’s capabilities and their motivation to treat clients effectively [50]. The suggestion put forward by DBT therapists that supervision be made available to teams in the longer term emphasises the value clinicians ascribed to expert supervision. As there were no DBT supervisors trained in Ireland at the start of this project in 2013, supervision was provided by international experts. This brings its own set of challenges, more specifically with regard to the outer setting CFIR construct; international supervisors would not have been familiar with local, cultural and service contexts in the Irish public health service. Given that DBT implementation in Ireland is still in its infancy, a sufficient volume of experienced clinicians who could offer such expert supervision is yet to be realised. However, a train-the-trainer model (e.g. [17]) could assist in developing and providing structures which would support clinicians working within the Irish health service and continue to practice DBT adherently.

### Inner and outer settings

Inner and outer setting factors that were important for successful DBT implementation included having sufficient resources and management commitment to support staff to train in and implement DBT. An unexpected finding from this study however, which corresponds to the outer setting domain in the CFIR, was in reference to eligibility criteria for referrals to the DBT programme. DBT team leaders in AMHS reported that referral agents on their teams were sometimes slow to assign a diagnostic label of BPD/EUPD to individuals who met criteria for same. This may have resulted in individuals who were suitable for the DBT intervention not being referred, or having a delayed referral to the programme. This finding is complex and requires further exploration as it is possible that it reflects historical, contextual and phenomenological factors whereby services anecdotally tended to avoid making a diagnosis of personality disorder in the absence of evidence-based assessment and therapeutic approaches. As a diagnosis of BPD is not typically given to adolescents under the age of 18 in Ireland, referral criteria in CAMHS instead focused on evidence of emotional and behavioural dysregulation. Consideration needs to be given to whether diagnostic criteria is necessary for referral into DBT when behavioural indicators or levels of dysregulation may be more effective in matching clients to the intervention.

While ‘planning’ and ‘engaging’ of the CFIR ‘Process’ construct were two critical steps carefully considered in the design phase of the coordinated implementation, some key challenges still arose. For example, in the ‘planning’ phase, teams interested in training via the NDBTPI were required to complete an application form which provided detail about the proposed DBT team and included management sign-off to indicate their awareness of the application and training requirements. After teams successfully secured a place to train, an orientation meeting then took place with the proposed DBT team, in addition to a meeting with the local management team, to build commitment and clarify how DBT would fit within the service goals [42, 44]. Despite this, over time, many participants described a stressful reality of being required to undertake DBT without any reduction or adaptation to their existing workload. This failure to reduce staff-held responsibilities has also been noted as a barrier to successful DBT implementation in other studies [17, 34]. While therapists were dedicated to the implementation of DBT and tried to manage DBT provision in addition to their typical workload, this strong commitment may not be sustainable in the long term. Other research has cautioned against an overreliance on the individual(s) involved in the intervention to ensure successful implementation [33]. These findings, together with work by Carmel et al. [17], highlight the potential for clinician burnout or practitioner turnover [33]. For mental health services providing DBT in community settings, an ongoing challenge will be the management of routine clinical work while also sustaining and scaling DBT provision to meet growing population need when additional resources may not be available. Given the continued movement of staff in addition to staff attrition, having ongoing training opportunities to replace and enhance existing DBT teams with the addition of new members was clearly identified by participants in this study as an important factor for sustainability. In addition to the provision of training, participants also highlighted the importance of supervision and support to provide guidance on effectively integrating new team members which will optimise and enhance service provision.

### Individuals involved/ process

Another domain of the CFIR refers to the individuals involved in the intervention or implementation process. Client selection emerged as a strong theme in this study. While appropriate recruitment of clients was identified as challenging in a previous study [17], the additional challenge of rushed recruitment and consideration for the requirement of a longer pre-treatment phase were highlighted in the results of this study. This could be a consequence of therapists in this setting working in a publicly funded under-resourced system or may also be attributed to the requirements of taking part in a coordinated implementation approach with suggested timelines for programme establishment and guidelines for programme requirements etc.

Numerous findings from this study point to the value of establishing a support forum for DBT teams. DBT team leaders suggested that linking with existing teams at the implementation preparation stage would be useful to identify potential implementation barriers and access resources. DBT therapists also noted that liaising with other DBT teams to meet and share ideas would be helpful. Formalising this support through the establishment of a DBT support forum may play a role in fostering sustainability. Additionally, team leaders also faced pressure to deliver DBT whilst simultaneously grappling with the challenge of leading the DBT team. The findings from this study make a strong case for establishing a forum where team leaders can support one another. This is an important point to consider given that having an effective team leader was cited by DBT therapists as a facilitator for DBT implementation. Based on these findings, the NDBTPI commissioned specific leader training through the licensed training provider to support DBT leaders in championing the DBT model and managing the challenges of both delivering an evidence-based intervention and stewarding colleagues towards adherent practice in the absence of holding a line management function. To this end, continuing to provide additional team leader training and potentially offering dedicated supervision hours for team leaders would likely be useful.

While staff selection for training has previously been identified as important [39], the theme of team formation in this study highlights the additional importance of considering team size at the planning stage. Team leaders from large DBT teams highlighted challenges with team dynamics, an uneven distribution of workload and time pressure in the weekly consultation meetings while smaller teams reported strong team dynamics. Given that having dedicated team members was reported as the main facilitator to implementation from the perspective of DBT therapists, these findings highlight the importance of not only considering staff selection for training, but also considering the impact of team size on the wider team. Coinciding with these findings, the licensed training providers have reduced the maximum team size from ten to eight.

### Limitations

There are limitations to this study which warrant consideration. The sample in this study consists of teams of clinicians who attended DBT training following an application process with a coordinating and support team. In particular, the first cohort of team leaders could be considered to be ‘early adopters’ and primed DBT champions. Therefore, there may be an over-representation of clinicians who are more highly motivated to implement a new evidence-based treatment for BPD in their service compared to other individuals who did not avail of the same early training opportunities.

Secondly, the response rate to the survey at the final data collection time-point was lower than anticipated, despite efforts by the research team to increase response rates. While the research team were aware that some attrition was inevitable given that some therapists had left either the DBT team or their local community mental health team by the end of the study, it is possible that the remaining therapists who did not respond may have more negative feedback. We were somewhat reassured however by the fact that there was representation from each team in the survey data.

Finally, the participants in this study received their training in one central location as a cohort of eight teams following a successful application to the NDBTPI. As funding for training and supervision was provided by the NDBTPI, there was less pressure for participants in this study to secure local financial investment for DBT. Recent research indicates that those receiving on-site training have poorer DBT team survival than those trained on open-enrolment training [33]. Teams presenting to open-enrolment training must secure local organisational support and financial investment to train. These pre-training actions by the team may shape organisational change in favour of sustainability [33]. Funding for training was therefore less of a consideration for participants in this study in comparison to other studies. However, local organisational support for funding resources was identified as an issue by some participants in this study.

### Future research

Future longitudinal research could consider how targeting interpersonal factors such as team cohesion, communication skills, ongoing provision of expert supervision and training content and format might impact on DBT implementation outcomes. Additionally, implementation hybrid study designs which compare a number of different types of implementation approaches are required to see which are most effective. For example, one approach might involve managers attending a one-day seminar on implementation, while another may focus on developing a service plan for treating clients and examining which of these approaches results in more effective implementation and sustainability.

## Conclusions

It is recommended, in future, that clinicians and health service managers consider the inclusion of a clear whole system mandate to provide a DBT intervention as a sustained part of service provision in a public health service. The lack of a mandate for the implementation of DBT led to unforeseen and difficult challenges in this study. While re-allocation of capacity and resources may facilitate the introduction of an evidence-based treatment such as DBT in the short term, long-term sustainability will be contingent on realistic levels of funding, resources and organisational support within the health system. Successful implementation is an iterative process. Key insights from the implementation of DBT with these 16 teams provide useful guidance to inform sustainability of existing teams and to scale service provision to achieve national coverage in the Irish public health system.

## Supplementary information

**Additional file 1.** Consolidated Framework for Implementation Research ‘Process’ domain: application of the Consolidated Framework for Implementation Research ‘Process’ domain to the National DBT Project Ireland.

**Additional file 2.** Implementation Process Survey: survey developed by research team to explore various aspects of the implementation process experienced by clinicians who trained via the National DBT Project Ireland.

## Data Availability

The dataset used for the survey analysis in this study are available from the corresponding author on reasonable request. The dataset used for the interview analysis is not publicly available due to the small number of participants and potential for identification of participants.

## Abbreviations

BPD: Borderline personality disorder
CFIR: Consolidated framework for implementation research
DBT: Dialectical behaviour therapy
NDBTPI: National DBT Project Ireland
NOSP: National Office for Suicide Prevention
AMHS: Adult Mental Health Services
CAMHS: Child and Adolescent Mental Health Services

## Footnotes

1 Intensive training (part one) comprises a five day teaching block in which teams are taught the principles of establishing a DBT programme and core strategies of the individual therapy component of the treatment. Part two typically takes place within 8 months of part one for which teams present their work on the programme and receive feedback and expert consultation on both their programme and individuals cases [39].

2 In order to protect confidentiality of participants and in an attempt to encourage honest responses, two researchers who conducted the interviews with team leaders had no direct contact or liaison with Cohort 1 teams prior to conducting the interviews.
