# Supplementary material for: Evaluating the national multisite implementation of dialectical behaviour therapy in a community setting: a mixed methods approach
Source: BMC Psychiatry. 2020 May 14;20:235. doi: 10.1186/s12888-020-02610-3 (PMC7227064; doi:10.1186/s12888-020-02610-3)
Supplement: Supplementary file 1 — Additional file 1. Consolidated Framework for Implementation Research ‘Process’ domain: application of the Consolidated Framework for Implementation Research ‘Process’ domain to the National DBT Project Ireland. [file 12888_2020_2610_MOESM1_ESM.docx]

**April – July 2013 (4 months)***

**July 2013 – July 2015 (24 months)**

**December 2013 – May 2014 (6 months)**

**July 2013 – March 2014 (9 months)**

*Process*

- Preparation of detailed protocol to evaluate the national coordinated implementation
- Review of outcomes and feedback from participants (service users, DBT therapists)
- Continued refinement of implementation process following feedback review
- Meeting with National Office for Suicide Prevention to discuss funding opportunities
- Securing funding
- Recruitment and appointment of coordinating team
- Appointment of Steering Group Committee
- Consideration and identification of training application process
- Teams attend Intensive Training-Part I
- Following attendance at training, contact from coordinating team to support implementation in service and identify programme start date for all teams
- Implementation of first DBT programme delivery in services
- Informing clinicians and managers about upcoming training opportunity and potential for DBT implementation
- Orientation meeting with teams that had been selected to attend DBT training
- Mandatory meetings between coordinating team and management teams in areas where teams had been selected to attend DBT training
- Individual team site visits with coordinating team and clinicians identified to attend training
- Meeting with treatment developer, Prof. Linehan, and key stakeholders (e.g. service users, managers)

*Timeline pertains to year one of the National DBT Project

*Flynn D, Kells M, Joyce M, Corcoran P, Gillespie C, Suarez C, Swales M, Arensman E. Innovations in Practice: Dialectical behaviour therapy for adolescents: multisite implementation and evaluation of a 16‐week programme in a public community mental health setting. Child and Adolescent Mental Health. 2019 Feb;24(1):76-83.*
