# Supplementary material for: Evaluating the national multisite implementation of dialectical behaviour therapy in a community setting: a mixed methods approach
Source: BMC Psychiatry. 2020 May 14;20:235. doi: 10.1186/s12888-020-02610-3 (PMC7227064; doi:10.1186/s12888-020-02610-3)
Supplement: Supplementary file 2 — Additional file 2. Implementation Process Survey: survey developed by research team to explore various aspects of the implementation process experienced by clinicians who trained via the National DBT Project Ireland. [file 12888_2020_2610_MOESM2_ESM.doc]

Date completed: ____________________________

National DBT Project - Feedback Form

|  |
| --- |

Please complete the following feedback form about your participation

in the National DBT Project over the past 2 years.

Please circle the response that is most applicable for you.

If any of the questions do not apply to you, please circle the *Not Applicable (N/A)* option.

Please do not write your name anywhere in this form.

**Experience of the coordinated implementation:**

1. Please rate your experience of how responsive the National DBT Project Office

have been with regard to the following:

Telephone Response:

| Poor | Fair | Good | Very good | N/A |
| --- | --- | --- | --- | --- |

Email Response:

| Poor | Fair | Good | Very good | N/A |
| --- | --- | --- | --- | --- |

Personal contact (e.g. at training days, data collection):

| Poor | Fair | Good | Very good | N/A |
| --- | --- | --- | --- | --- |

2. In your opinion, is there additional information or assistance that could be provided

by the National DBT Project Office to help teams with the implementation of a DBT

programme in their area?

Yes _ _ No _ _

If yes, please elaborate:

--------------------------------------------------------------------------------------------------------------------------------------------------------------------------------------------------------------------------------------------------------------------------------------------------------------------------------------------------------------------------------------------------------------------------------------------------------------------

----------------------------------------------------------------------------------------------------------------------------------------------------------------------------------------------------------------------------------

3. In your opinion, is there additional training that could be provided to help you and/or

your DBT team with **long-term sustainability** of DBT in your service?

Yes _ _ No _ _

If yes, please specify what kind of training you think would be helpful:

--------------------------------------------------------------------------------------------------------------------------------------------------------------------------------------------------------------------------------------------------------------------------------------------------------------------------------------------------------------------------------------------------------------------------------------------------------------------

4. What specific things (other than additional training) do you think would be helpful for

you and/or your DBT team to help with **long-term sustainability** of DBT in your

service?

--------------------------------------------------------------------------------------------------------------------------------------------------------------------------------------------------------------------------------------------------------------------------------------------------------------------------------------------------------------------------------------------------------------------------------------------------------------------

**Supervision:**

5. Please rate your experience of the following practical aspects of DBT supervision:

Ease of establishing initial contact with DBT supervisor:

| Poor | Fair | Good | Very good | N/A |
| --- | --- | --- | --- | --- |

Responsiveness of DBT supervisor to queries between supervision sessions:

| Poor | Fair | Good | Very good | N/A |
| --- | --- | --- | --- | --- |

Scheduling of supervision sessions/keeping scheduled supervision appointments:

| Poor | Fair | Good | Very good | N/A |
| --- | --- | --- | --- | --- |

Please give examples to support your ratings:

--------------------------------------------------------------------------------------------------------------------------------------------------------------------------------------------------------------------------------------------------------------------------------------------------------------------------------------------------------------------------------------------------------------------------------------------------------------------

6. Please indicate how helpful DBT supervision has been in your DBT practice with

regard to the following:

Programme Elements (Group Skills/Individual Therapy/Phone Coaching/Consult):

| Not helpful | A little helpful | Somewhat helpful | Very helpful | N/A |
| --- | --- | --- | --- | --- |

Structuring the Environment/Service Related Issues:

| Not helpful | A little helpful | Somewhat helpful | Very helpful | N/A |
| --- | --- | --- | --- | --- |

Please give examples to support your ratings:

--------------------------------------------------------------------------------------------------------------------------------------------------------------------------------------------------------------------------------------------------------------------------------------------------------------------------------------------------------------------------------------------------------------------------------------------------------------------

7. In your opinion, has your supervisor:

Actively encouraged you to work with 2+ DBT clients: Yes _ _ No _ _

Encouraged you to record sessions for review: Yes _ _ No _ _

Please elaborate:

--------------------------------------------------------------------------------------------------------------------------------------------------------------------------------------------------------------------------------------------------------------------------------------------------------------------------------------------------------------------------------------------------------------------------------------------------------------------

8. In your opinion, was the supervision resource, which was made available to you

through the National DBT Project Office, sufficient to meet your needs?

Yes _ _ No _ _

If no, please provide further details:

--------------------------------------------------------------------------------------------------------------------------------------------------------------------------------------------------------------------------------------------------------------------------------------------------------------------------------------------------------------------------------------------------------------------------------------------------------------------

9. In your opinion, is there additional support that could be provided regarding

**supervision** opportunities to help you and/or your DBT team with **long-term**

**sustainability** of DBT in your service?

Yes _ _ No _ _

If yes, please specify what kind of support you think would be helpful:

--------------------------------------------------------------------------------------------------------------------------------------------------------------------------------------------------------------------------------------------------------------------------------------------------------------------------------------------------------------------------------------------------------------------------------------------------------------------

-----------------------------------------------------------------------------------------------------------------

**Foundational Training:**

(Foundational Training is a five-day training for clinicians who wish to join an existing

Intensively Trained DBT Team)

10. Was there sufficient demand for DBT resources in your service to warrant the

addition of new therapists to your DBT team?

Yes _ _ No _ _

Please elaborate:

--------------------------------------------------------------------------------------------------------------------------------------------------------------------------------------------------------------------------------------------------------------------------------------------------------------------------------------------------------------------------------------------------------------------------------------------------------------------

-----------------------------------------------------------------------------------------------------------------

11. Did your DBT team apply for Foundational Training through the National DBT

Project Office?

Yes _ _ No _ _

If no, what barriers prevented your team from applying for Foundational Training?

--------------------------------------------------------------------------------------------------------------------------------------------------------------------------------------------------------------------------------------------------------------------------------------------------------------------------------------------------------------------------------------------------------------------------------------------------------------------

-----------------------------------------------------------------------------------------------------------------

12. Was there sufficient staff interest locally to train the permitted maximum of four

additional therapists at Foundational Training?

Yes _ _ No _ _

Please elaborate:

--------------------------------------------------------------------------------------------------------------------------------------------------------------------------------------------------------------------------------------------------------------------------------------------------------------------------------------------------------------------------------------------------------------------------------------------------------------------

13. Following the addition of new members to your DBT team at Foundational Training,

are there sufficient resources now available to meet local service demands?

Yes _ _ No _ _ N/A _ _

If no, please elaborate:

--------------------------------------------------------------------------------------------------------------------------------------------------------------------------------------------------------------------------------------------------------------------------------------------------------------------------------------------------------------------------------------------------------------------------------------------------------------------

14. Please outline how the addition of new therapists has impacted upon your DBT team

(e.g. increased capacity to meet service demand for DBT/challenges associated with

having a larger DBT team):

--------------------------------------------------------------------------------------------------------------------------------------------------------------------------------------------------------------------------------------------------------------------------------------------------------------------------------------------------------------------------------------------------------------------------------------------------------------------

-----------------------------------------------------------------------------------------------------------------

15. Following the addition of new therapists to your DBT team, what additional

supports, if any, would be helpful to facilitate the integration of new team members?

--------------------------------------------------------------------------------------------------------------------------------------------------------------------------------------------------------------------------------------------------------------------------------------------------------------------------------------------------------------------------------------------------------------------------------------------------------------------

-----------------------------------------------------------------------------------------------------------------

**Experience of DBT Implementation:**

16. Please rate your experience of implementing DBT in your service with regard to the

following:

HSE Local Management Support (e.g. Line Manager):

| Poor | Fair | Good | Very good |
| --- | --- | --- | --- |

HSE Area Management Support (e.g. ECD, Operations Manager):

| Poor | Fair | Good | Very good |
| --- | --- | --- | --- |

Support from Psychiatrist/Referral Agents:

| Poor | Fair | Good | Very good |
| --- | --- | --- | --- |

Support from non-DBT colleagues on Community Mental Health Team:

| Poor | Fair | Good | Very good |
| --- | --- | --- | --- |

17. Please identify one thing that **assisted** in the successful implementation of DBT in

your service:

-------------------------------------------------------------------------------------------------------------------------------------------------------------------------------------------------------------------------------------------------------------------------------------------------------------------------------------------------------------------------------------------------------------------------------------------------------------------------------------------------------------------------------------------------------------------------------------

-----------------------------------------------------------------------------------------------------------------

18. Please identify one **barrier** to the successful implementation of DBT in your service:

-------------------------------------------------------------------------------------------------------------------------------------------------------------------------------------------------------------------------------------------------------------------------------------------------------------------------------------------------------------------------------------------------------------------------------------------------------------------------------------------------------------------------------------------------------------------------------------

-----------------------------------------------------------------------------------------------------------------

19. On average, how much time per week do you spend on DBT related work:

| 0.5 days | 1 day | 1.5 days | 2 days |
| --- | --- | --- | --- |

Other (please specify):

----------------------------------------------------------------------------------------------------------------

----------------------------------------------------------------------------------------------------------------

20. Please write any additional comments in the space below:

(regarding your experience of training in DBT, implementing DBT in your service, participating in a coordinated implementation project etc.)

Thank you for your participation
